# Age at smoking initiation and self-rated health among second grade high school boys and girls in Scania, Sweden, a cross-sectional study

**DOI:** 10.1186/s12889-015-2457-z

**Published:** 2015-11-18

**Authors:** Kristina Hansen, Martin Lindström, Maria Rosvall

**Affiliations:** Department of Clinical Sciences, Social Medicine and Health Policy, CRC, Scania University Hospital, Lund University, Jan Waldenströms gata 35, SE-205 02 Malmö, Sweden

**Keywords:** Self-rated health, Smoking initiation, Smoking onset, Life-course

## Abstract

**Background:**

Smoking is usually initiated early in life and most adult regular smokers have started smoking before 18 years of age. A younger age at smoking initiation is associated with risk taking behaviours and worse health outcomes regarding psychological and somatic conditions, suggested to be caused by exposure during critical developmental periods. The present study aims to investigate self-rated health among second grade high school boys and girls related to age at smoking initiation (<14 years of age and ≥ 14 years of age) among current and former smokers, compared to never smokers.

**Methods:**

Data was derived from the Scania public health survey among children and adolescents in 2012. The study was cross-sectional with retrospective information about first time cigarette smoking experiences among 3245 boys and 3434 girls in second grade of high school. Self-rated health was assessed with the question “How do you rate your general health”. Associations of age at smoking initiation, current smoking status and poor self-rated health were investigated with logistic regression models.

**Results:**

Crude odds ratios of poor self-rated health were increased for all smoking groups compared to never smokers. Former smoking boys and currently smoking girls with early smoking initiation had the highest odds ratios of poor self-rated health, with odds ratios (OR) 2.4 (95 % confidence interval (CI): 1.5–3.7) and OR 2.9 (95 % CI: 2.3–3.6), respectively. After adjustments for sociodemographic factors, health-related behaviours, psychosocial factors, weight and functional disabilities, the results were attenuated, but remained statistically significant regarding former and current smoking boys with early smoking initiation, OR 2.0 (95 % CI: 1.1–3.7) and OR 1.7 (95 % CI: 1.1–2.4) and for current smoking girls with early and later smoking initiation, OR 2.1 (95 % CI: 1.5–2.8) and OR 1.5 (95 % CI: 1.1–2.0).

**Conclusion:**

Boys and girls in second grade of high school with early smoking initiation reported poorer self-rated health than later initiators and never smokers. Poorer self-rated health persisted also after smoking cessation among early initiating boys. Further studies are needed to understand the adverse health effects associated with timing of smoking initiation.

## Background

Smoking is usually initiated early in life and most adult regular smokers have started smoking before 18 years of age [1, 2]. In several European countries, 70 % or more of adult former and current smokers started smoking regularly before the age of 18 years [3]. Globally smoking prevalences among the young vary and WHO report on prevalences between 8–21 % among boys and 2–17 % among girls [4]. Early smoking initiation, before 14 years of age, is more prevalent among boys and vary considerably between countries e.g., 56 % in Estonia and 9 % in Armenia initiate smoking before 14 years of age [5].

Smoking in Sweden has declined among men since the 1970s and among women since the 1980s [6], however, in younger age groups, 16–24 years of age, smoking has been fairly consistent over the last 10 years [7]. Among Swedish second grade high school students, 10 % of the boys and 14 % of the girls report daily or almost daily smoking and additionally 16 % of the boys and 19 % of the girls report intermittent smoking. In the same group 19 % of both boys and girls report smoking before the age of 14 years [8].

Studies report more subjective physical and psychological health complaints, poorer self-rated health and lower quality of life already among adolescents associated with daily smoking, intermittent smoking and ex-smoking compared to never smokers [9–14] suggesting that smoking has a prompt connection to health.

Early adolescence is a vulnerable period in life where major physical and emotional changes are initiated including physical growth, sexual maturation, hormone changes, development of identity and mental and social development [15, 16]. Smoking initiation during early compared to later adolescence has been associated with increased risk taking behaviours such as substance use [17], alcohol risk consumption [18], sexual risk behaviours [13] and suicidal behaviours [19]. Earlier smoking onset compared to later has further been shown to be associated with more unfavourable physical health outcomes several years after smoking initiation, even after controlling for total smoking exposure. Such an effect has been suggested to act through various pathways e.g., by modulating immune system response [20–22] causing DNA damage, diminishing organ growth [23], or initiating atherosclerotic development [24, 25], with more severe effects from tobacco smoke exposure during periods of growth and development [20, 23].

Furthermore, neurobiological studies suggest that early smoking has an unfavourable effect due to the ongoing brain development [2], where early smoking onset compared to later could increase the risk of long standing adverse health effects more than expected from only prolonged smoking exposure. Younger age at smoking onset, compared to later has been associated with psychological symptoms such as attention deficit hyperactivity disorder (ADHD), shorter time to onset of first anxiety disorder and earlier and more frequent episodes of major depressive disorders [17, 26–28].

Self-rated health (SRH) is a well-established measure among adults found to be a reliable outcome of physical and psychological wellbeing and a predictor of mortality [29–31]. Among adolescents self-rated health is as a measure of physical and psychological wellbeing that also include “an overall sense of function” [32, 33]. As clinical endpoints in these age groups are sparse, self-rated health might be a suitable measure of health in adolescents and young adults.

Early smoking initiation might have critical impact on future health development concerning physical, psychological and behavioural health effects, independent of smoking duration. Identifying effects related to age at smoking initiation is important when trying to understand the development of health pathways and to guide future public health efforts. To the best of our knowledge no previous study has explored the association of early and later smoking initiation, current smoking status and SRH in adolescence. In the present study we aim to investigate self-rated health among second grade high school boys and girls related to age at smoking initiation (<14 years of age and ≥ 14 years of age) and current smoking status.

## Methods

### Study design

The study is cross-sectional with retrospective information about first time cigarette smoking experiences among 3245 boys and 3434 girls in second grade of high school in 2012.

### Study population

This study was based on The Scania public health survey among children and adolescents conducted in 2012, addressing all 33 municipalities in the county of Scania. The study population has previously been described in detail elsewhere [34]. In short: Among pupils in the 6th and 9th grade of elementary school and second grade of high school self-reported anonymous questionnaires covering school conditions, health issues, leisure time habits, food habits, alcohol, tobacco and drug habits, were distributed and gathered in the classroom by teachers, during school hours. In all, almost 30 000 pupils answered the survey questionnaire. Participants were informed about the survey purpose, their anonymity, their optional participation, confidentiality of answers and that the results would be used in research. Parents of the participating children and adolescents were likewise informed. Written parental consent was not required for the present study, as second grade high school students are considered mature enough to by themselves decide on participation in this type of public health survey in Sweden.

*Inclusion criteria*; the current study is a secondary study based on all 9987 of 13 848 eligible pupils in second grade high school answering the questionnaire, yielding a response rate of 72 %. *Exclusion criteria;* subjects with missing data on sex (*n* = 139), conflicting, invalid or missing responses of smoking status (*n* = 923) as well as subjects that did not smoke but had tried (*n* = 2246) were excluded and reduced the study population to 3245 boys and 3434 girls and these subjects were born between 1991–1995.

### Definitions

#### Dependent variable

*Self-rated health* was assessed with the question “How do you rate your general health” with optional answers being very good, good, neither good nor poor, poor and very poor. The answers were dichotomized into *poor self-rated* health including neither good nor poor, poor and very poor self-rated health and *good self-rated* health including good and very good self-rated health.

#### Independent variables

*Smoking status* was assessed with two questions; 1: “Do you smoke?” with the optional answers: “No, I have never smoked”; “No, but I have tried”; “No, I have smoked but stopped”; “Yes, every day”; “Yes, almost every day”; “Yes, when attending parties” and “Yes, sometimes” and question 2: “How old were you when (if ever) you smoked a cigarette?”. Subjects reporting “No, I have smoked but stopped” were considered former smokers. Subjects were classified as current smokers if reporting daily smoking, almost daily smoking, smoking when attending parties or smoking sometimes. Former and current smokers were divided into early and later smoking initiators with a cut-off at 14 years of age. Subjects stating “No, I don’t smoke but I have tried” were excluded from the analyses due to their rather low use of cigarettes.

#### Covariates

*Parental country of birth* was divided into: both parents born in Sweden, at least one parent born in Sweden and both parents born abroad. *Parental employment* was divided into: both parents working, one parent working and no parent working.

The variable *intense alcohol consumption* was constructed based on how often quantities of alcohol equivalent to 25 cl hard liquor were consumed during the last 12 months. Examples were given in different standard containers. Subjects reporting drinking these quantities at least once a month were considered intense alcohol consumers [35]. *Use of narcotic drugs* was assessed with the question: “Have you used at least one narcotic drug during the last 12 months?” with optional answers, no-I have never used narcotics, no-I have not used narcotics during the last 12 months, yes-hash/marijuana, yes-ecstasy, yes-amphetamine, yes-other narcotics and answers were dichotomized into: No, including I have never used narcotics and no, not during the last 12 months and the remaining alternatives were dichotomized as Yes.

*Not easy to talk to parents or friends if problems* was assessed with the question “If you have any problems or just want to talk to someone how easy or difficult do you think it is to turn to parents or grown ups with whom you live” or “If you have any problems or just want to talk to someone how easy or difficult do you think it is to turn to friends,” respectively. The optional answers were: “very easy”, “rather easy”, “neither easy nor difficult”, “rather difficult” and “very difficult”. Answers were dichotomized into easy to talk to parents or friends, respectively, including the two first alternatives and not easy to talk to parents or friends, respectively, including the three latter alternatives.

*Weight categories* were constructed by calculating Body Mass Index (BMI) based on self-reported weight and height with four categories; underweight; BMI < 18.5, normal weight; BMI 18.5–24.9, overweight; BMI 25–29.99 and obesity; BMI ≥ 30 [36].

*Functional disability* was assessed with the question: “Do you have any of following disabilities? hearing disability, visual disability that cannot be corrected with glasses or lenses, physical disability, reading-writing-dyslexia-disability, Attention Deficit Hyperactivity Disorder (ADHD) or Attention Deficit Disorder or other disability?” A dichotomous variable was constructed grouping subjects with at least one functional disability compared to none.

### Statistical analyses

**Fig. 1:**
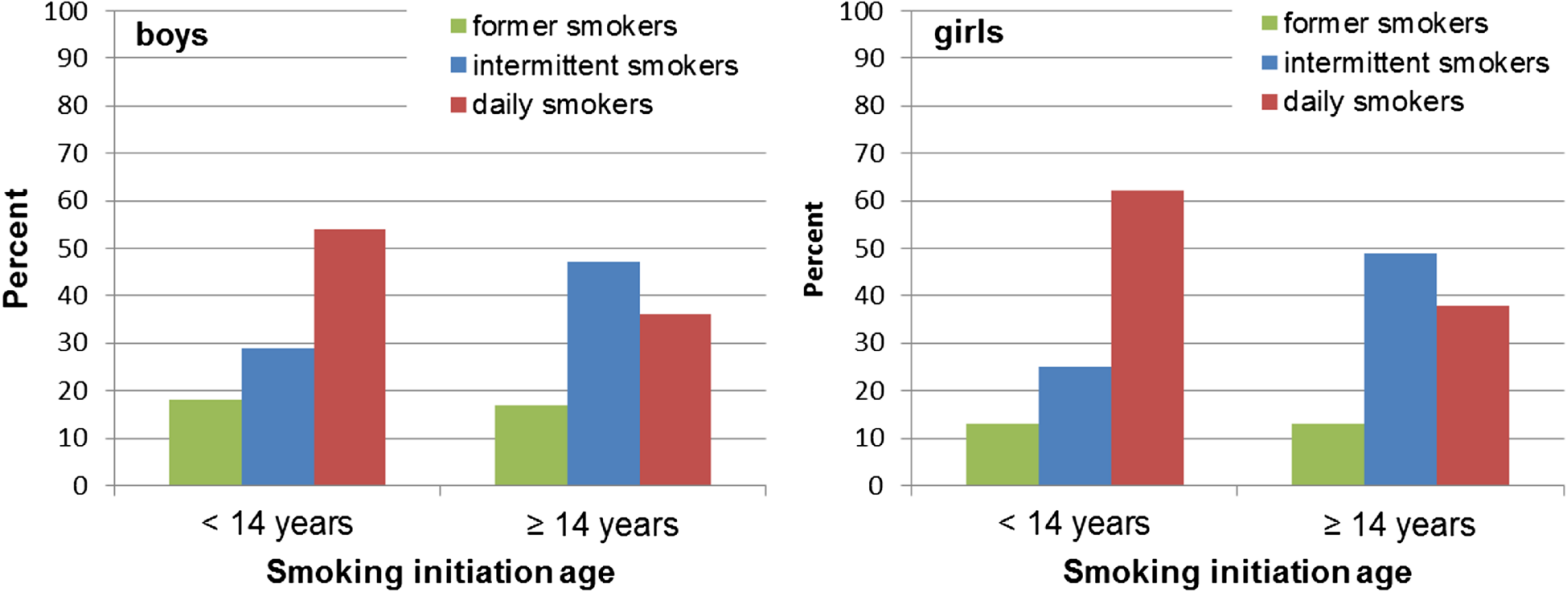
Current smoking status among second grade high school students by age of smoking initiation. Distribution of current smoking status related to early or later smoking initiation among second grade high school boys and girls

The study population was stratified by sex and divided into five groups; never smokers, former smokers initiating smoking before 14 years of age, current smokers initiating smoking before 14 years of age, former smokers initiating smoking at 14 years of age or older and current smokers initiating smoking at 14 years of age or older. The cut-off at 14 years of age was based on previous studies [35]. Study population characteristics related to smoking status are presented in Tables 1 and 2. Current smoking status by early or late smoking initiation is presented in Fig. 1.

**Table 1:** Characteristics (%) of high school boys by smoking status and age at smoking initiation

	Boys *n* = 3245				
		Early initiators^a^		Later initiators^b^	
	Never smokers	Former smokers	Current smokers	Former smokers	Current smokers
	*n* = 1467 (45 %)	*n* = 136 (4 %)	*n* = 640 (20 %)	*n* = 166 (5 %)	*n* = 836 (26 %)
Sociodemographic factors (%)					
Parental country of birth					
Both parents born in Sweden	70	75	67	63	70
One parent born in Sweden	11	10	14	17	12
Both parents born abroad	19	15	19	19	18
Parental employment					
Both parents working	82	79	75	78	83
One parent working	15	17	17	19	15
No parent working	3	5	8	3	3
Health related lifestyle factors (%)					
Intense alcohol consumption^c^	17	64	73	55	67
Narcotic drugs last 12 months^d^	3	32	42	24	29
Psychosocial factors (%)					
Not easy to talk to parents if problems	32	42	42	47	41
Not easy to talk to friends if problems	28	26	24	20	22
Health related symptoms (%)					
Weight^e^					
Underweight	8	3	5	8	5
Normal weight	76	66	71	68	73
Overweight	14	24	18	15	17
Obesity	2	7	7	9	6
Functional disability^f^	21	39	39	41	28
Poor self-rated health	11	23	20	13	15

Characteristic (%) of second grade high school boys by smoking status and age at smoking initiation, the Scania public health survey among children and adolescents, 2012

^a^Early initiators-initiating smoking <14 years of age

^b^Later initiators-initiating smoking ≥ 14 years of age

^c^Intense alcohol consumption-consumtion of alcohol equivalent to 25 cl hard liquor at least once a month during the last 12 months

^d^Narcotic drugs-used at least one narcotic drugs during last 12 months

^e^Weight; underweight-Body Mass Index (BMI) <18.5, normal weight-BMI 18.5–24.9, overweight-BMI 25–29.99, obesity-BMI ≥30

^f^Functional disability-reporting at least one disability-hearing disability, visual disability that cannot be corrected with glasses or lenses, physical disabilities, reading-writing-dyslexia-disabilities, Attention Deficit Hyperactivity Disorder or Attention Deficit Disorder or other disability

**Table 2:** Characteristics (%) of high school girls by smoking status and age a smoking initiation

	Girls *n* = 3434				
		Early initiators^a^		Later initiators^b^	
	Never smokers	Former smokers	Current smokers	Former smokers	Current smokers
	*n* = 1613 (47 %)	*n* = 89 (3 %)	*n* = 594 (17 %)	*n* = 144 (4 %)	*n* = 994 (29 %)
Sociodemographic factors (%)					
Parental country of birth					
Both parents born in Sweden	70	70	67	70	71
One parent born in Sweden	10	13	16	11	12
Both parents born abroad	21	18	17	18	17
Parental employment					
Both parents working	81	83	74	80	81
One parent working	15	13	21	18	15
No parent working	4	4	5	2	4
Health related lifestyle factors (%)					
Intense alcohol consumption^c^	11	43	65	43	57
Narcotic drugs last 12 months^d^	1	14	32	15	20
Psychosocial factors (%)					
Not easy to talk to parents if problems	30	45	44	42	39
Not easy to talk to friends if problems	20	30	23	22	19
Health related symptoms (%)					
Weight^e^					
Underweight	14	20	11	14	10
Normal weight	75	63	73	73	79
Overweight	9	13	13	11	8
Obesity	2	5	3	3	3
Functional disability^f^	19	25	35	30	26
Poor self-rated heath	15	25	33	21	23

Characteristics (%) of second grade high school girls by smoking status and age at smoking initiation, the Scania public health survey among children and adolescents, 2012

^a^Early initiators-initiating smoking <14 years of age

^b^Later initiators-initiating smoking ≥14 years of age

^c^Intense alcohol consumption-consumtion of alcohol equivalent to 25 cl hard liquor at least once a month during the last 12 months

^d^Narcotic drugs-used at least one narcotic drugs during last 12 months

^e^Weight; underweight-Body Mass Index (BMI) < 18.5, normal weight-BMI 18.5–24.9, overweight-BMI 25–29.99, obesity-BMI ≥30

^f^Functional disability-reporting at least one disability-hearing disability, visual disability that cannot be corrected with glasses or lenses, physical disabilities, reading-writing-dyslexia-disabilities, Attention Deficit Hyperactivity Disorders or Attention Deficit Disorder or other disability

Logistic regression was used to analyse the association between smoking initiation age period, current smoking status and self-rated health with the results presented in Table 3. Odds ratios of poor self-rated health and 95 % confidence intervals were calculated and presented in a crude model and four additional models adjusted for covariates; model 1: adjusted for age, parental country of birth and parental employment, model 2: additionally adjusted for intense alcohol consumption and use of at least one narcotic drug during the last 12 months, model 3: additionally adjusted for not easy to talk to parents if problems and not easy to talk to friends if problems and model 4: additionally adjusted for weight and presence of functional disability. All statistical analyses were performed with IBM SPSS statistics version 22.

**Table 3:** Odds ratios and 95 % CI of poor self-rated health of early and later smoking initiators

	Poor self-rated health									
	Crude	CI	Model 1^a^	CI	Model 2^b^	CI	Model 3^c^	CI	Model 4^d^	CI
Boys										
Never smokers	1		1		1		1		1	
Early initiators former smokers	2.4	1.5–3.7	2.1	1.3–3.4	2.2	1.3–3.6	1.9	1.1–3.3	2.0	1.1–3.7
Early initiators current smokers	2.0	1.6–2.6	1.9	1.4–2.5	1.8	1.3–2.5	1.7	1.2–2.4	1.7	1.1–2.4
Later initiators former smokers	1.3	0.8–2.0	1.1	0.7–1.9	1.0	0.6–1.8	0.9	0.5–1.7	0.9	0.5–1.6
Later initiators current smokers	1.5	1.1–1.9	1.5	1.1–1.9	1.4	1.1–2.0	1.3	0.9–1.8	1.4	1.0–2.0
Girls										
Never smokers	1		1		1		1		1	
Early initiators former smokers	2.0	1.2–3.3	1.7	1.0–3.0	1.7	1.0–3.1	1.5	0.8–2.8	1.5	0.8–2.9
Early initiators current smokers	2.9	2.3–3.6	2.6	2.1–3.3	2.4	1.8–3.1	2.1	1.6–2.9	2.1	1.5–2.8
Later initiators former smokers	1.6	1.03–2.4	1.5	1.0–2.4	1.5	1.0–2.4	1.3	0.8–2.2	1.3	0.7–2.1
Late initiators current smokers	1.8	1.4–2.2	1.7	1.4–2.1	1.6	1.3–2.1	1.5	1.2–1.9	1.5	1.1–2.0

Crude and adjusted odds ratios (OR) and 95 % confidence interval (CI) of poor self-rated health by early (< 14 years) or late (≥ 14years) smoking initiation and current smoking status among second grade high school students, stratified by sex. The Scania public health survey among children and adolescents, 2012

^a^Model 1-adjusted for age, parental country of birth and parental employment

^b^Model 2-adjusted for model 1 and intense alcohol consumption and use of narcotic drugs last 12 months

^c^Model 3-adjusted for model 2 and not easy to talk to parents and not easy to friends if problems

^d^Model 4-adjusted for model 3 and weight and functional disability

The study was approved by the Ethical Committee at Lund University, Sweden (Dnr. no. 2013/317).

## Results

In the final study population, 1467 (45 %) were never smokers, 302 (9 %) former smokers and 1476 (45 %) current smokers among boys (Table 1). Out of the ever smoking boys 43 % were early initiators and 56 % were later initiators. Among girls 1613 (47 %) were never smokers, 233 (7 %) former smokers, and 1588 (46 %) current smokers (Table 2). Out of the ever smoking girls 38 % were early smoking initiators and 62 % were later smoking initiators.

Table 1 presents characteristics of the study population by current smoking status and age at smoking initiation (<14 years of age and ≥14 years of age) among second grade high school boys. Early initiating former smokers showed higher prevalences of having both parents born in Sweden, intense alcohol consumption, use of narcotic drugs, not easy to talk to friends if problems, overweight and poor self-rated health and lower prevalences of not easy to talk to parents if problems than later initiating former smokers. Early initiating current smokers showed higher prevalences of intense alcohol consumption, use of narcotic drugs, at least one disability and poor self-rated health and lower prevalences of both parents working than later initiating current smokers.

Table 2 presents characteristics of the study population by current smoking status and age at smoking initiation (<14 years of age and ≥14 years of age) among second grade high school girls. Early initiating former smokers had higher prevalences of not easy to talk to friends if problems, underweight and poor self-rated health and lower prevalence of at least one disability than later initiating former smokers. Early initiating current smokers showed higher prevalences of intense alcohol consumption, use of narcotic drugs, not easy to talk to parents if problems, not easy to talk to friends if problems, overweight, at least one disability and poor self-rated and lower prevalences of both parents born in Sweden and both parents working than later initiating current smokers.

Figure 1 presents the distribution of current smoking status in relation to early or later smoking initiation among boys and girls. Among those who started smoking before the age of 14 the proportion of daily smokers was higher than among those with a later smoking onset in both boys (*p* < 0.05) and girls (*p* < 0.05). The proportions of former smokers were approximately the same in both early and later smoking onset groups. In the groups with later smoking initiation, the proportions of intermittent smokers were higher than among early initiators (boys *p* < 0.05, girls *p* < 0.05).

Table 3 presents crude and adjusted odds ratios of poor self-rated health by age at smoking initiation and current smoking status among second grade high school students stratified by sex. In the crude model ORs of poor self-rated health were increased for all smoking groups compared to never smokers, most markedly for former and current smokers with early smoking initiation among both boys and girls. After adjustments for age, parental country of birth, parental employment, intense alcohol consumption, use of at least one narcotic drug during the last 12 months, not easy to talk to parents if problems, not easy to talk to friends if problems, weight and functional disability in the final model, the increased OR of poor self-rated health were attenuated, but remained statistically significant for former and current smoking boys with early smoking initiation, OR = 2.0 (95 % CI: 1.1–3.7) and OR = 1.7 (95 % CI: 1.1–2.4) and current smoking girls with early and later smoking initiation, OR = 2.1 (95 % CI: 1.5–2.8) and OR = 1.5 (95 % CI: 1.1–2.0).

## Discussion

Boys and girls in the second grade of high school with early smoking initiation (before 14 years of age) generally reported poorer self-rated health than later initiators and never smokers. Poorer self-rated health persisted also after smoking cessation among early initiating boys.

Adolescence is a growth period that marks the transition from childhood to adulthood, and can be divided into early 10–13 years, middle 14–16 years and late 17–19 years adolescence based on stages of development [15]. During early adolescence major physical and emotional changes are initiated including physical growth, sexual maturation, hormone changes, development of identity, mental and social development [15, 16]. These changes will continue to progress during mid adolescence and are completed in late adolescence [15]. Development periods are considered vulnerable to external exposure as cell proliferation and differentiation are accelerated and mutagenic alterations can appear [37]. Theories suggest that earlier smoking initiation during a vulnerable development period could have more severe health effects than later smoking initiation [2, 23]. Neurobiological studies report that early smoking initiation might affect brain development by modulating glutaminergic, dopaminergic and serotonergic systems and further affect emotional life, intellectual capacity and reactions to drugs [2, 38]. Previous studies have also shown associations between early initiation of smoking and poor psychological health such as depression, anxiety, and ADHD [17, 26–28]. However, results are inconclusive concerning the temporal directions. A younger compared to older initiation age has further been associated with chronic disease independently of the extent of prior tobacco exposure with regard to cardiovascular disease [24], peripheral vascular diseases [25], squamous cell carcinoma of the cervix [20] and lung cancer [21, 22].

In the present study, early compared to later initiating currently smoking boys and girls less often had both parents working. A previous review study supports these findings and report on earlier smoking initiation in lower socioeconomic groups [39]. In a national survey from the US early smoking initiation was associated with low socioeconomic status based on education, employment and income [18].

The present study showed higher prevalences of intense alcohol consumption, use of narcotics and daily smoking among early smoking initiators compared to later initiators. Such higher smoking intensity might theoretically have more serious effects on the developing adolescent body compared to less intense smoking. A younger age at smoking initiation has also in previous studies been linked to risk taking behaviors such as alcohol use [18], drug use, [17] suicidal [19], sexual risk behaviors [13] and transition to daily smoking [40].

The results of the present study imply that poor health in late adolescence is connected to early smoking initiation. Such poor self-rated health persisted even after smoking cessation among early initiating boys. This might indicate that smoking initiation in young ages is connected with a persistent effect on health even after cessation. Furthermore, possibly those with the strongest effects on health due to smoking quit to a higher degree than those experiencing less health effects.

Early inequalities in health need to be addressed promptly and at the relevant time span to maximize individual and public health benefits. Prevention efforts directed towards young adolescents should emphasise short-term effects, but also consider the possible long-term effects of smoking exposure during extra vulnerable periods in life. Comprehending long-term effects of smoking such as cancer and chronic obstructive pulmonary disease is possibly difficult in younger ages. Using self-rated health, effects on health associated with smoking that emerge already among adolescents are made visible. Public health strategies aimed to hinder smoking initiation are the most important, but also efforts aimed at supporting smoking cessation need to start at an early age.

## Strengths and limitations

Self-rated health is considered a reliable and valid measure among early-and middle adolescents and young adults [33] and is an internationally used measure associated with morbidity and mortality [30, 31]. Furthermore, the data material allows for adjustments for potential confounders, however, it is not possible to fully exclude residual confounding. Socioeconomic status is associated with SRH health in adolescence and adulthood [12, 32, 41, 42] and could possibly influence our investigated associations. We have adjusted for socioeconomic status as parental employment, but it is possible that this variable might not fully account for the effect of socioeconomic status. Almost all adolescents in Sweden attend high school [43], making it possible to reach a high proportion of adolescents through class-room surveys. The proportion of students smoking before 14 years of age in the study group was approximately the same as in national studies and around 19 % [8].

A limitation in the present study is the cross-sectional design with retrospective information about age at smoking initiation. The lack of information on self-rated health at the time of smoking initiation makes it hard to ascertain the temporal direction in the association between smoking onset and SRH later in adolescence. Furthermore, it is difficult to distinguish effects of age at smoking onset from effects of smoking duration and thus a cumulative health effect from smoking. Moreover, risks of recall bias and reporting bias are problems connected to self-reported measures. However, the fact that the questionnaire was anonymous and no connections to individuals could be made enhances the possibilities of truthful reporting. Furthermore, second grade high school students are considered mature enough to by themselves decide on participation in this type of public health survey in Sweden. Validation of self-reported smoking has in previous studies shown satisfactory agreement concerning report of never, current and former smoking status [44, 45]. In the main analyses we chose to exclude subjects that did not smoke but had tried smoking due to its theoretically smaller health effects. However, in an additional analysis combining never smokers and those who tried smoking, we found similar patterns of increased adjusted odds ratios (AOR) of poor self-rated health among early initiating former smoking boys, AOR = 2.0 (95 % CI:1.1–3.6), early initiating currently smoking boys, AOR =1.6 (95 % CI:1.2–2.3), early initiating former smoking girls AOR = 1.4 (95 % CI:0.7–2.7) and early initiating currently smoking girls AOR = 1.9 (95 % CI:1.5–2.6) as in the main analysis. Complete information on smoking status was missing on 923 students who were excluded from the analyses. Students with such missing information showed no consistent association with unfavourable life style factors compared to the group with complete data on smoking in that they less often reported intense alcohol consumption, but more often reported overweight or obesity. Furthermore, there were no differences in SRH between the two groups.

Post hoc sample size calculations generally showed good enough power to detect differences in SRH between investigated smoking groups and never smokers in both boys and girls, however, with low power in the group later initiators former smokers. Given a larger sample size in this group might result in statistically significant differences in SRH also in boys.

## Conclusion

Boys and girls in the second grade of high school with early smoking initiation generally reported poorer self-rated health than later initiators and never smokers. Such poor self-rated health persisted also after smoking cessation among early initiating boys. Further studies are needed to understand the adverse health effects associated with the timing of smoking initiation.

## Abbreviations

ADHD: Attention deficit hyperactivity disorder
AOR: Adjusted odds ratios
BMI: Body mass index
CI: Confidence interval
OR: Odds ratio
SRH: Self-rated health
